# Critical role of IL-25-ILC2-IL-5 axis in the production of anti-*Francisella* LPS IgM by B1 B cells

**DOI:** 10.1371/journal.ppat.1009905

**Published:** 2021-08-27

**Authors:** Carlos Henrique D. Barbosa, Louis Lantier, Joseph Reynolds, Jinyong Wang, Fabio Re

**Affiliations:** Department of Microbiology and Immunology, Rosalind Franklin University of Medicine and Science, North Chicago, Illinois, United States of America; University of Maryland, Baltimore, UNITED STATES

## Abstract

B1 cells, a subset of B lymphocytes whose developmental origin, phenotype, and function differ from that of conventional B2 cells, are the main source of “natural” IgM but can also respond to infection by rapidly producing pathogen-specific IgM directed against T-independent antigens. *Francisella tularensis* (*Ft*) is a Gram-negative bacterium that causes tularemia. Infection with *Ft* Live Vaccine Strain activates B1 cells for production of IgM directed against the bacterial LPS in a process incompletely understood. Here we show that immunization with purified *Ft* LPS elicits production of LPS-specific IgM and IgG_3_ by B1 cells independently of TLR2 or MyD88. Immunization, but not infection, generated peritoneum-resident memory B1 cells that differentiated into LPS-specific antibody secreting cells (ASC) upon secondary challenge. IL-5 was rapidly induced by immunization with *Ft* LPS and was required for production of LPS-specific IgM. Antibody-mediated depletion of ILC2 indicated that these cells were the source of IL-5 and were required for IgM production. IL-25, an alarmin that strongly activates ILC2, was rapidly secreted in response to immunization or infection and its administration to mice significantly increased IgM production and B1 cell differentiation to ASC. Conversely, mice lacking IL-17RB, the IL-25 receptor, showed impaired IL-5 induction, IgM production, and B1 ASC differentiation in response to immunization. Administration of IL-5 to *Il17rb*^*-/-*^ mice rescued these B1 cells-mediated responses. *Il17rb*^*-/-*^ mice were more susceptible to infection with *Ft* LVS and failed to develop immunity upon secondary challenge suggesting that LPS-specific IgM is one of the protective adaptive immune mechanisms against tularemia. Our results indicated that immunization with *Ft* LPS triggers production of IL-25 that, through stimulation of IL-5 release by ILC2, promotes B1 cells activation and differentiation into IgM secreting cells. By revealing the existence of an IL-25-ILC2-IL-5 axis our results suggest novel strategies to improve vaccination against T-independent bacterial antigens.

## Author summary

B1 cells are a subset of B lymphocytes that participate in the immune response to infection by producing antibodies of the IgM class. Here we investigate the mechanisms that control B1 cells activation and production of IgM directed against the lipopolysaccharide (LPS) of *Francisella tularensis*, a Gram-negative bacterium that causes tularemia. Using a mouse model of tularemia, our results revealed that *Francisella* LPS elicits production of the cytokine IL-25 that in turn activates blood cells called Innate Lymphoid Cells 2 (ILC2). Once activated, ILC2 produce the cytokine IL-5 that is required for activation of B1 cells and production of IgM. Mice unresponsive to IL-25 are more susceptible to *F*. *tularensis* infection. By revealing the existence of an IL-25-ILC2-IL-5 axis our results suggest novel strategies to improve vaccination against bacteria.

## Introduction

Antibodies are among the most effective mechanisms that protect us against pathogens. IgM is the first immunoglobulin class to be produced during an infection and, because of its high avidity, agglutination capacity, and ability to activate complement, it plays a critical role in the early phase of the infection. The existence of non class-switched IgM memory B cells suggests that this antibody is important during the secondary response as well [1–3]. IgM can be divided into two classes- natural IgM and immune IgM (reviewed in [4]). Natural IgM is constitutively produced regardless of presence of antigen or infection, is polyreactive, tends to recognize self-antigens, phospholipid, or capsular carbohydrates, and provides protection against certain infections [5,6]. It is primarily produced by B1 B cells, a subset of B cells that resides mainly in the pleural and peritoneal cavity and that differs from the classical B2 cells for development, phenotype, and function [7,8]. B1 cells are also capable to rapidly produce pathogen-specific immune IgM directed against T-independent (TI) antigens such as capsular polysaccharides and microbial glycolipids and, therefore, B1 cells represent a first line of defense against several pathogens. Activation and differentiation of B1 cells in response to infection is understood to a much lower degree than that of classical B2 cells. Peritoneal B1 do not secrete IgM at steady state or following challenge [9–11]. Rather, in response to bacterial infections, B1 cells migrate from body cavities to the spleen and lymphoid organs where they differentiate into antibody secreting cells (ASC). Pathogen-specific memory B1 cells that provide long-term protection has also been reported [12–15]. Based on expression of CD5, B1 cells can be divided into the B1a (CD5^+^) and B1b (CD5^-^) subsets. Although it has been proposed that B1a cells specialize in production of natural antibodies while B1b cells are devoted to production of pathogen-specific IgM [16], a number of studies that investigated activation of B1 cells in response to bacterial and viral infections indicate a more complex scenario. B1a cells were shown to respond to influenza virus [17,18] and *Francisella tularensis* Live Vaccine Strain (LVS) [14,19,20] whereas B1b cells produced antigen-specific IgM against *Borrelia hermsii* [12,13,21], *Salmonella typhimurium* [8,22], *Streptococcus pneumoniae* [16,23] and the filarial nematode *Litomosoides sigmodontis* [24]. It has also been shown that *Mycobacterium tuberculosis* lipid antigen can trigger differentiation of both B1a and B1b cells into ASC [25]. Interestingly, during respiratory infections with the influenza virus or the filarial nematode B1 cells accumulated in the respiratory lymph nodes and pleural cavity but not in the spleen, thus restricting the production of IgM to just the lung environment.

Innate lymphoid cells (ILC) are a heterogeneous family of lymphocytes that lack antigen receptors and contribute to both innate and adaptive immunity by producing a number of cytokines in response to infection or tissue stress [26]. Depending on the cytokines they preferentially produce, ILC are divided into various subsets. Relevant to this study, the ILC2 subset is enriched at mucosal sites, particularly the lung, and secretes large amount of type 2 cytokines, including IL-5 and IL-13, when stimulated by alarmins like IL-25 (IL-17E) or IL-33 [27].

*Francisella tularensis* is a gram-negative bacterium that causes tularemia, a highly lethal disease particularly in the pneumonic form [28]. *F*. *tularensis* and the attenuated *F*. *tularensis* LVS (hereafter *Ft*) infect myeloid cells and other non-phagocytic cells, escape the phagosome, and replicate in the cytoplasm. Innate immune detection of *Ft* is mediated primarily by recognition of bacterial lipoprotein by TLR2 [29–32] and the AIM2 inflammasome [33]. Interestingly, *Ft* possesses an atypical LPS that does not stimulate TLR4 and lacks pro-inflammatory activity [34–36], a feature that likely contributes to this bacterium’s virulence. In this regard, *Ft* LPS should be considered as a TI type 2 antigen, due to its polymeric structure, rather than a TI type 1, like the TLR-stimulating LPS of most Gram-negative bacteria. Several innate and adaptive immune effector mechanisms participate in the protective response to infection with *Ft* including phagocytes, NK cells, IgG, IgA, and various subsets of T and B cells [37,38]. We and others have previously shown that IgM specific for *Ft* LPS is rapidly produced by B1a cells and that passive immunization with anti-*Ft* LPS IgM is protective against *Ft* LVS intranasal infection [14,20]. The inability of *Ft* LPS to stimulate TLR or an inflammatory response raises the question of the mechanism responsible for its activation of B1 cells and the role of innate immune pathways in this process. Here we report that immunization with purified *Ft* LPS elicited production of antigen-specific IgM by B1 cells independently of TLR2 or MyD88. Immunization, but not infection with *Ft*, generated a population of peritoneal IgM memory B1 cells. Importantly, we demonstrate that B1 activation by *Ft* LPS is controlled by an IL-25-ILC2-IL-5 axis.

## Results

### Production of *Ft* LPS-specific IgM is MyD88- and TLR-independent

**Fig 1:**
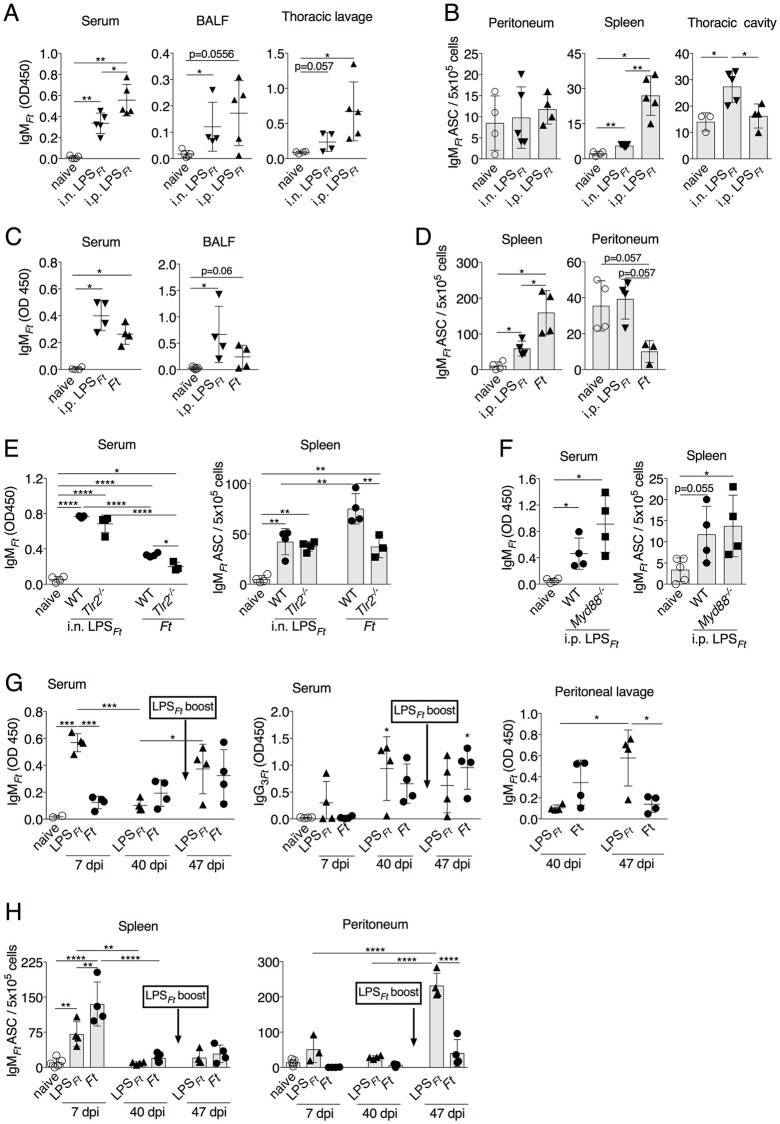
LPS_*Ft*_ elicits production of IgM_*Ft*_ independently of TLR2 or MyD88 and induces IgM memory B1 cells. (A-F) WT B6, *Tlr2*^*-/-*^ or *Myd88*^*-/-*^ mice were immunized intranasally (i.n.) or intraperitoneally (i.p.) with LPS_*Ft*_ or infected i.n. with *Ft* (4x10^3^ cfu). Seven days post-immunization IgM_*Ft*_ was measured in serum, BALF, or thoracic cavity lavage (A, C, E, F) and IgM_*Ft*_ ASC were enumerated by ELISPOT (B, D, E, F). (G, H) Groups of WT B6 mice were immunized i.p. with LPS_*Ft*_ or infected i.n. with *Ft* (4x10^3^ cfu) and euthanized at the shown time points for measurement of IgM_*Ft*_ or IgG_3*Ft*_ in serum and peritoneal lavage and enumeration of IgM_*Ft*_ ASC. On day 40 post-immunization, groups of mice received an i.p. LPS_*Ft*_ booster shot and were euthanized seven days later (day 47). One representative experiment of five (A, D), two (E-H) is shown. Data are expressed as mean ± SD. A-D, F, Mann-Whitney U test; E, G, H one-way ANOVA Tukey Post-test. *p<0.05, **p<0.01, ***p<0.001, ****p<0.0001. See also S1 Fig.

*Ft* infection or immunization with purified *Ft* LPS (hereafter, LPS_*Ft*_) stimulate production of IgM specific for LPS_*Ft*_ (hereafter, IgM_*Ft*_) by B1 cells [14,19,20]. LPS_*Ft*_ is unable to stimulate TLR or induce an inflammatory response and yet it is strongly immunogenic [39]. To better understand this process, mice were immunized with LPS_*Ft*_, either intranasally (i.n.) or intraperitoaneally (i.p.) (Fig 1A and 1B), or i.n. infected with *Ft* LVS (Fig 1C and 1D). Seven days later, IgM_*Ft*_ was measured in serum, BALF, or thoracic cavity lavage. Both immunization routes with purified LPS_*Ft*_ induced production of IgM_*Ft*_ but the i.p. route consistently yielded higher IgM_*Ft*_ serum titers and numbers of ASC in spleen. Total IgM levels were not affected by immunization/infection (S1 Fig). Immunization with as little as 25 ng of LPS_*Ft*_ was sufficient to stimulate this response (S1 Fig). We confirmed that our preparation of LPS_*Ft*_ was devoid of pro-inflammatory activities and that IgM_*Ft*_ did not cross-react with LPS extracted from other Gram-negative bacteria (not shown). After encounter with antigen, B1 cells migrate to the spleen where they differentiate into ASC, a process shown to be regulated by TLR stimulation [40]. In agreement with this, the ELISPOT number of IgM_*Ft*_ ASC found in the spleen on day 7 was significantly higher in *Ft*-infected mice, where TLR stimulation occurs, compared to mice immunized with “TLR-inactive” LPS_*Ft*_ (Fig 1B and 1D). The increased migration of B1 cells to spleen in *Ft*-infected mice was paired to a decreased number of IgM_*Ft*_ ASC in the peritoneal cavity (Fig 1D). The total number of B1 cells in spleen and peritoneum was not changed by immunization (S1 Fig).

Our previous work showed that production of IgM_*Ft*_ during intranasal *Ft* infection depends on IL-1β and TLR2 signaling [20]. In contrast, immunization with purified LPS_*Ft*_ elicited comparable levels of IgM_*Ft*_ in WT mice and in *Tlr2*^*-/-*^ or *Myd88*^*-/-*^ mice (Fig 1E and 1F), consistent with the TLR-inactive nature of LPS_*Ft*_. Thus, in the context of infection, TLR and IL-1β signaling contributes to IgM_*Ft*_ production, but during immunization Myd88-mediated signaling appears dispensable. As previously shown [19], this response was T-independent (not shown).

### Immunization, but not infection, generates peritoneal IgM memory B1 cells

The IgM_*Ft*_ serum level was maximal on day 7 post-immunization and started decreasing thereafter, though it remained above the pre-immune level for an extended time (Fig 1G). Forty days after immunization/infection, mice received a LPS_*Ft*_ booster shot and were euthanized 7 days later to enumerate IgM_*Ft*_ ASC and measure IgM_*Ft*_ serum levels. As shown in Fig 1G, IgM_*Ft*_ returned to the level observed in the primary immunization. Interestingly, in both experimental groups the number of spleen IgM_*Ft*_ ASC did not increase upon challenge (Fig 1H), as observed during the primary response. Instead, in the immunized, but not the infected mice, the majority of IgM_*Ft*_ ASC were found in the peritoneal cavity. The level of IgM_*Ft*_ was also significantly increased in the peritoneum of the immunized mice (Fig 1G). These results indicate that immunization generates a population of IgM memory B1 cells that resides in the peritoneal cavity and that can rapidly differentiate into ASC when re-stimulated by antigen independently of input from the splenic environment, a phenomenon previously reported in different infection models [12,13,15,16,41]. Extrafollicular Ig class switch to the IgG_3_ isotype is known to occur in B1 cells [19,42]. LPS_*Ft*_ -specific IgG_3_ were not present in serum on day 7 p.i. but became detectable on day 40 (Fig 1G). Taken together, these results show that purified LPS_*Ft*_ strongly elicits IgM_*Ft*_ production and B1 cells differentiation into ASC independently of TLR and generates peritoneal IgM memory B1 cells.

### Both B1a and B1b cells can produce IgM_*Ft*_

**Fig 2:**
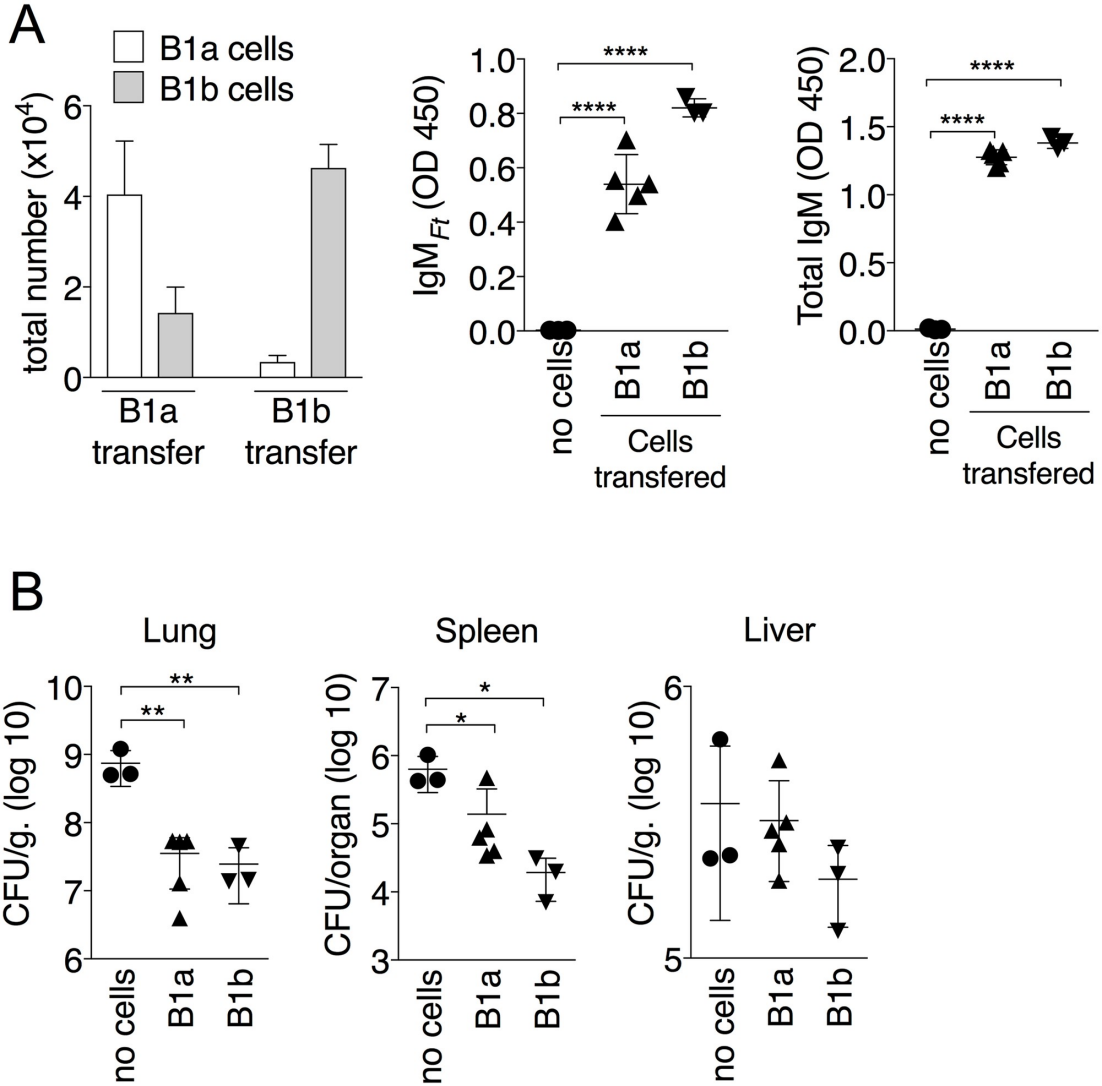
Both B1a and B1b cells produce IgM_*Ft*_. *Rag1*^*-/-*^ mice reconstituted with purified B1a or B1b cells were immunized i.p. with LPS_*Ft*_. (A) Total number of peritoneal B1a or B1b cells in reconstituted *Rag1*^*-/-*^ mice. Level of IgM_*Ft*_ and total IgM in serum was measured on day 7 p.i. (B) Reconstituted *Rag1*^*-/-*^ mice were then infected with *Ft* (4x10^3^ cfu) and euthanized seven days later for measurement of bacteria burdens in organs. One representative experiment of two is shown. Data are expressed as mean ± SD. One-way ANOVA Tukey Post-test. *p<0.05, **p<0.01, ****p<0.0001. See also S2 Fig.

Two subsets of B1 cells can be identified based on surface expression of the CD5 molecule. Previous work from our and others labs have shown that B1a cells produce IgM_*Ft*_ [14,19,20]. To better define the response to immunization with LPS_*Ft*_, B1a and B1b cells were purified from the peritoneal cavity of naïve WT mice by cell sorting and adoptively transferred i.p. into *Rag1*^*-/-*^ mice. Four weeks later, mice were immunized with purified LPS_*Ft*_ and production of IgM_*Ft*_ was measured in serum seven days after immunization. As shown in Fig 2A, mice reconstituted with B1b harbored mostly B1b cells in the peritoneal cavity. In contrast, both B1a and B1b cells were present in mice reconstituted with B1a. Whether this is due to higher plasticity of the B1a subset or to higher fitness of contaminating B1b cells present in small quantity in the B1a cells preparation is presently unclear. Regardless of the B1 subsets they received, both groups of mice produced IgM_*Ft*_ at comparable levels (Fig 2A). Interestingly, the level of IgM_*Ft*_ appeared to correlate with the number of B1b cells suggesting that this subset may be the one mainly responsible for production of IgM_*Ft*_. This is in agreement with the observation that B1b can respond to antigenic stimulation while B1a, which express the inhibitory CD5, are unresponsive [16,43]. In fact, a recent paper that examined the response to the TI-Ag NP-Ficoll concluded that B1b cells exhibited increased Ag receptor signaling, Blimp1 expression, and capacity to differentiate into ASC and memory cells compared to other B cells subsets including marginal zone and follicular B cells [44]. Seven days post-immunization, the reconstituted mice were then intranasally infected with *Ft* and euthanized seven days later. As shown in Fig 2B, bacteria burdens in lung and spleen were significantly lower in mice reconstituted with either B1 subset compared to non-reconstituted mice, in agreement with our previous observation that IgM_*Ft*_ are protective against *Ft* infection [20]. These results confirm the T-independent nature of IgM_*Ft*_ and suggest that both B1 subsets have the potential to respond to LPS_*Ft*_ immunization.

### IL-5 promotes IgM_*Ft*_ production by B1 cells

**Fig 3:**
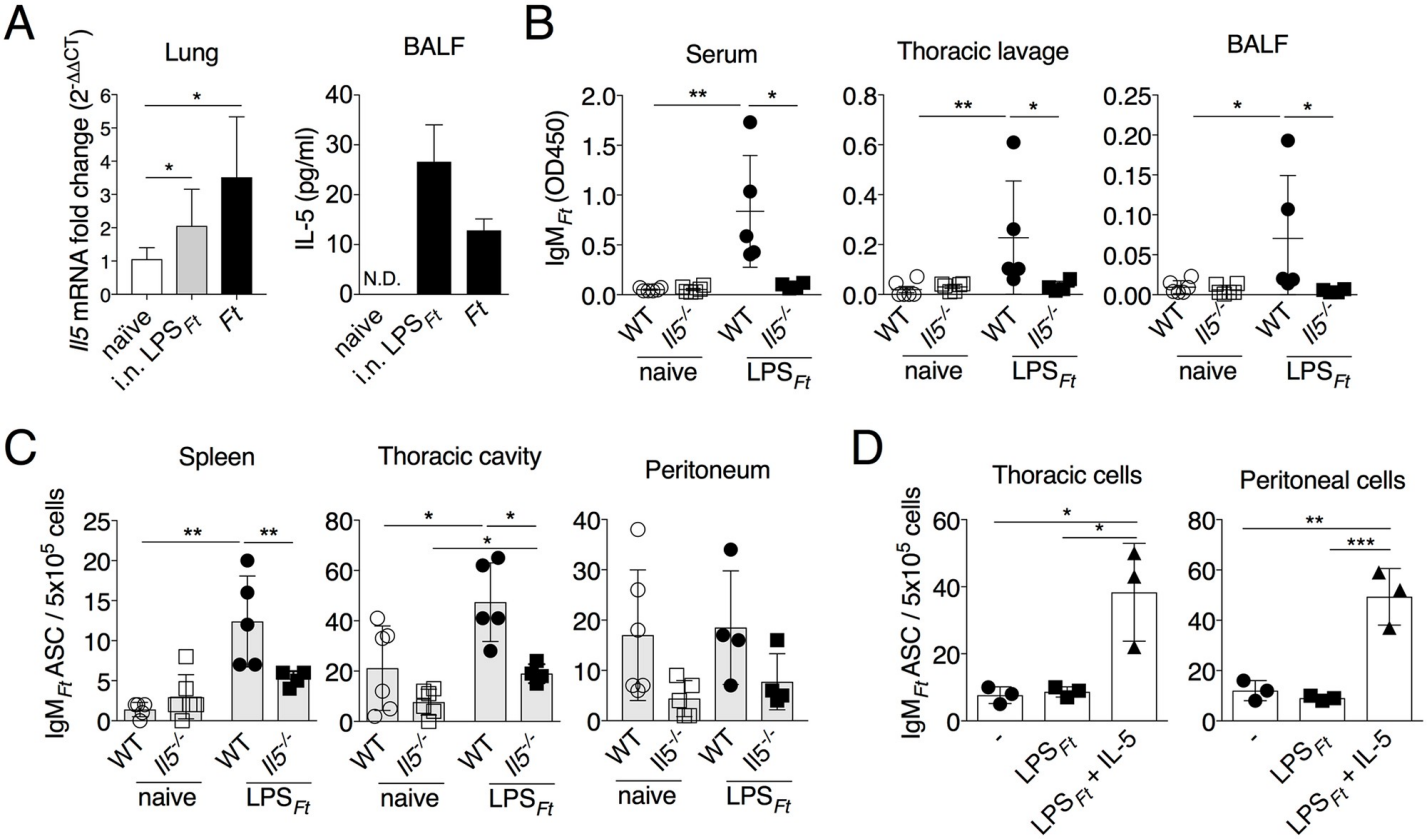
IL-5 promotes IgM_*Ft*_ production by B1 cells. (A) WT B6 mice (n = 3–8) were immunized i.n. with LPS_*Ft*_ or infected i.n. with *Ft*. Eighteen hrs p.i. *Il5* mRNA was measure in total lung RNA by RT-PCR and IL-5 protein in BALF by ELISA. (B, C) WT B6 or *Il5*^*-/-*^ mice were immunized with LPS_*Ft*_. Seven days post-immunization IgM_*Ft*_ was measured in serum, BALF, or thoracic cavity lavage and IgM_*Ft*_ ASC were enumerated by ELISPOT. (D) Cells purified from peritoneal or thoracic cavity of i.n. immunized WT B6 mice (day 7) were cultured in vitro with LPS_*Ft*_ in presence or absence of IL-5 for 2 days and then used to seed ELISPOT assay. One representative experiment of three (A) or two (B-D) is shown. Data are expressed as mean ± SD. A-C, Mann-Whitney U test; D, one-way ANOVA Tukey Post-test. *p<0.05, **p<0.01, ***p<0.001. See also S3 Fig.

IL-5 is a critical factor for development of B1 cells and has been shown to promote IgM production by B1 cells [45–48]. Importantly, although LPS_*Ft*_ does not stimulate TLR and lacks proinflammatory properties, IL-5 was rapidly induced after immunization with purified LPS_*Ft*_ (Fig 3A) suggesting a role for this cytokine in the IgM_*Ft*_ production by B1 cells. In support of this, immunized *Il5*^*-/-*^ mice produced significantly lower amount of IgM_*Ft*_ (Fig 3B) and had decreased number of IgM_*Ft*_ ASC in spleen and thoracic cavity compared to WT mice (Fig 3C). The total IgM levels and numbers of B1 cells and ILC2 were comparable in WT and *Il5*^*-/-*^ mice (S3 Fig). To test the ability of IL-5 to support IgM_*Ft*_ production independently of the effect this cytokine has on B1 cells development, B cells isolated from the peritoneal or thoracic cavity of WT mice seven days post-immunization were cultured in presence or absence of recombinant IL-5. As shown in Fig 3D significantly more IgM_*Ft*_ ASC were generated in presence of IL-5. These results show a critical role for IL-5 in production of IgM_*Ft*_ by B1 cells.

### ILC2 are critical for IgM_*Ft*_ production by B1 cells

**Fig 4:**
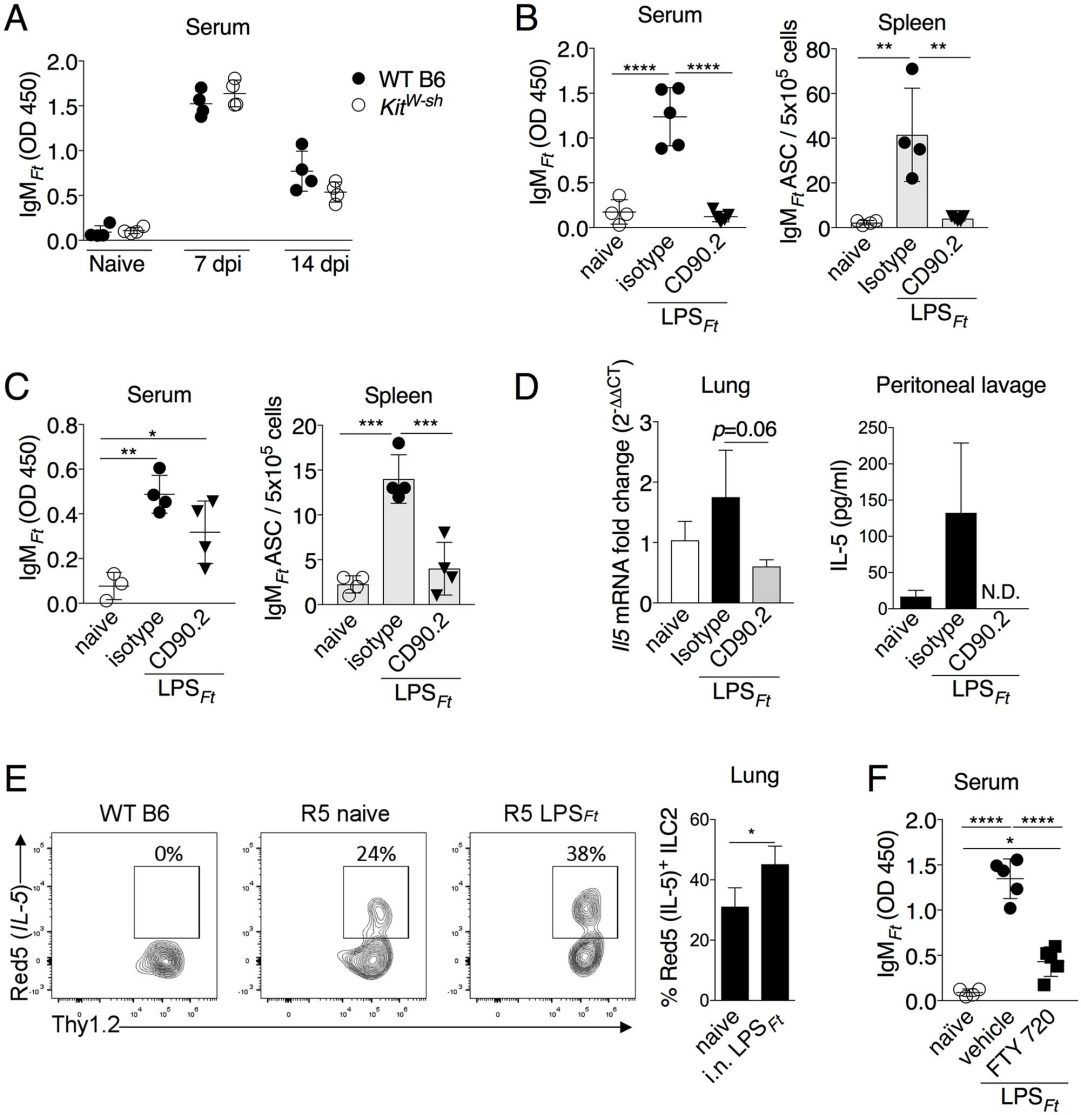
ILC2 are the source of IL-5 and are critical for IgM_*Ft*._ production by B1 cells. (A) Level of IgM_*Ft*_ in serum of i.p. LPS_*Ft*_-immunized WT B6 or mast cells-deficient *Kit*^*W-sh*^ mice on day 7 p.i. (B) *Rag1*^*-/-*^ mice reconstituted with WT peritoneal B cells or WT B6 mice (C) were depleted of ILC2 by i.p. injection of anti-CD90.2 or isotype Ab and immunized i.n. with LPS_*Ft*_. Level of IgM_*Ft*_ in serum and IgM_*Ft*_ ASC in spleen were measured on day 7 p.i. (D) *Il5* mRNA in lung and IL-5 protein in peritoneal wash were measured in WT B6 mice (n = 3) 18 hrs post-immunization (i.n.) with LPS_*Ft*_. (E) Red5 (*Il5*^*-/-*^) mice (n = 3) were immunized with LPS_*Ft*_ i.n. and expression of the reporter tdTomato was examined by FACS 18 hrs later in the KLRG1^+^ ILC2. (See S4 Fig). (F) WT B6 mice were treated with FTY720 and immunized with LPS_*Ft*_. IgM_*Ft*_ in serum was measured on day 7 p.i. One representative experiment of two or three (F) is shown. Data are expressed as mean ± SD. A-D, Mann-Whitney U test; E, unpaired students t test; F one-way ANOVA Tukey Post-test. *p<0.05, **p<0.01, ***p<0.001, ****p<0.0001. See also S4 Fig.

IL-5 is produced by both innate and adaptive immune cells, most prominently by T_h_2 cells, ILC2, and mast cells. The T-independent nature of IgM_*Ft*_ production suggested that either ILC2 or mast cells could be the source of IL-5 in immunized mice. Immunization of mast cell-deficient mice (*Kit*^*W-sh*^) elicited IgM_*Ft*_ level comparable to WT mice (Fig 4A) pointing to a critical role for ILC2. In order to test this hypothesis, *Rag1*^*-/-*^ mice, previously reconstituted with peritoneal B cells, were depleted of ILC2 by treatment with anti-CD90.2 antibody (S4 Fig) and were immunized with LPS_*Ft*_. As shown in Fig 4B, IgM_*Ft*_ serum level and number of IgM_*Ft*_ ASC in spleen were significantly reduced in ILC2-depleted mice. The numbers of ASC in the peritoneal and thoracic cavities were not affected by ILC2 depletion (S4 Fig). Similar results were obtained by depleting ILC2 in C57BL/6J mice (Fig 4C). Total IgM and numbers of B1 cells were not affected by ILC2 depletion (S4 Fig). ILC2-depletion also impaired immunization-induced *Il5* mRNA expression (Fig 4D) suggesting that ILC2 are indeed the source of IL-5. To test this hypothesis we took advantage of the Red5 mouse strain that expresses a tdTomato fluorescent reporter under the control of the *Il5* promoter [49]. As shown in Fig 4E, the percentage of KLRG1^+^ ILC2 expressing the reporter was increased by immunization with LPS_*Ft*_. Further supporting ILC2 role in IgM_*Ft*_ production, inhibition of S1P signaling, which is known to regulate migration of lymphocytes including ILC2 [50,51], significantly reduced IgM_*Ft*_ (Fig 4F). These results indicate that inflammatory ILC2 are necessary for B1 cells responses to LPS_*Ft*_ immunization and are the main source of IL-5.

### IL-25 enhances production of IgM_*Ft*_

**Fig 5:**
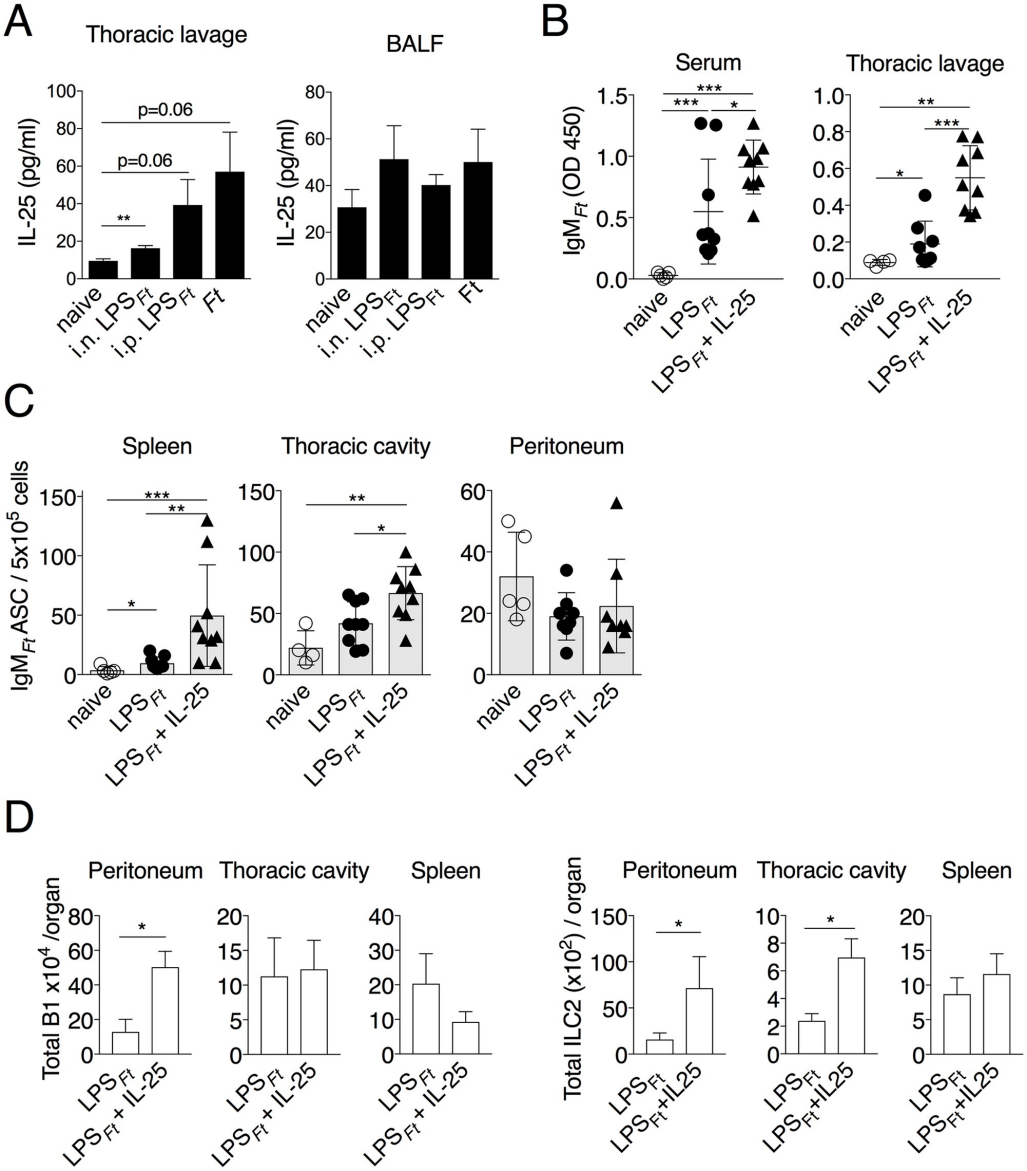
IL-25 enhances production of IgM_*Ft*_. (A) IL-25 was measured in BALF or thoracic cavity lavage 18 hrs post immunization or infection in WT B6 mice (n = 3). (B, C) WT B6 mice were treated with recombinant IL-25 and immunized i.n. with LPS_*Ft*_. Seven days p.i. IgM_*Ft*_ was measured in serum or thoracic cavity lavage and IgM_*Ft*_ ASC were enumerated by ELISPOT. (D) Total number of B1 and ILC2 cells. One representative experiment of two (A) or three (B, C) is shown. Data are expressed as mean ± SD. A, unpaired students t test; B-D, Mann-Whitney U test. *p<0.05, **p<0.01, ***p<0.001. See also S5 Fig.

IL-25 (IL-17E) and IL-33 are released by various cell types, including epithelial and stromal cells, in response to tissue stress and are among the most powerful activators of ILC2 [26]. The fact that production of IgM_*Ft*_ was not impaired in absence of Myd88, the adaptor used by the IL-33 receptor, suggested that this cytokine does not contribute to activation of B1 cells by LPS_*Ft*_. Therefore, we focused our attention on IL-25. IL-25 levels were rapidly increased in thoracic cavity and BALF of i.n. immunized or infected mice (Fig 5A). Administration of recombinant IL-25 to WT mice significantly increased IgM_*Ft*_ production and numbers of IgM_*Ft*_ ASC in spleen and thoracic cavity of LPS_*Ft*_ immunized mice (Fig 5B and 5C). An IL-25-dependent increase in number of peritoneal B1 was observed (Fig 5D) but did not translate into higher number of peritoneal ASC, likely due to the fact that the LPS_*Ft*_-specific cells migrated to the spleen. This treatment also significantly increased the number of ILC2 in peritoneum and thoracic cavity.

**Fig 6:**
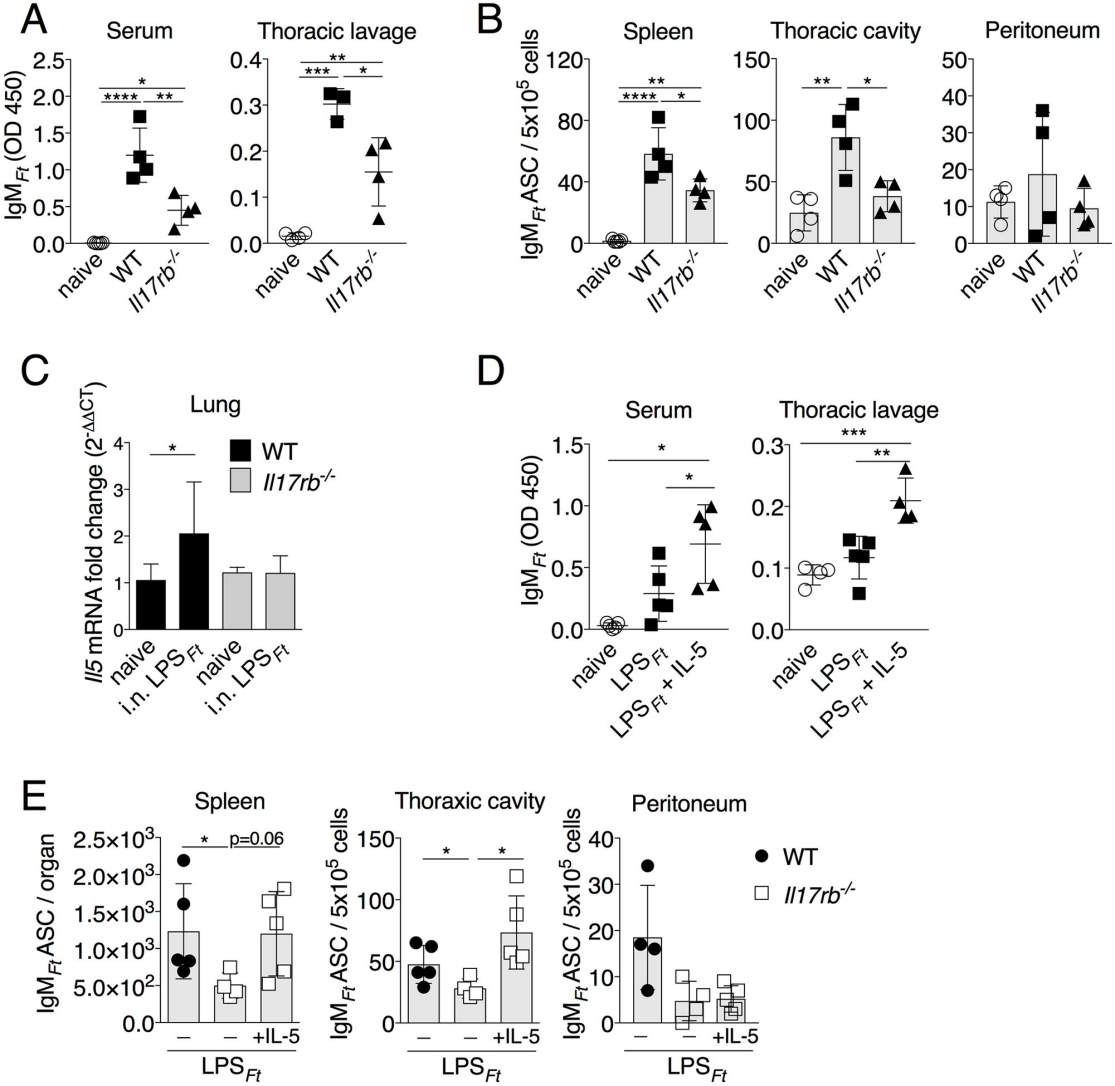
IL-17RB is required for IgM_*Ft*_ and IL-5 production. (A, B) WT B6 or *Il17rb*^*-/-*^ mice were immunized i.p. with LPS_*Ft*_. IgM_*Ft*_ in serum and thoracic lavage and IgM_*Ft*_ ASC in spleen, thoracic cavity and peritoneum were measured on day 7. (C) *Il5* mRNA was measure in total lung RNA of WT B6 or *Il17rb*^*-/-*^ mice (n = 3–8) 18 hrs p.i. (D, E) *Il17rb*^*-/-*^ mice were treated with IL-5 and immunized i.p. with LPS_*Ft*_. IgM_*Ft*_ and IgM_*Ft*_ ASC in spleen and thoracic cavity were measured on day 7. One representative experiment of three (A, B) or two (C) is shown. Data are expressed as mean ± SD. A-D, one-way ANOVA Tukey Post-test; E, Mann-Whitney U test. *p<0.05, **p<0.01, ***p<0.001, ****p<0.0001. See also S6 Fig.

To confirm the role of IL-25 in the production of IgM_*Ft*_, mice deficient in IL17RB [52], the IL-25 receptor, were immunized with LPS_*Ft*_. As shown in Fig 6A and 6B, level of IgM_*Ft*_ and number of IgM_*Ft*_ ASC were significantly reduced in immunized *Il17rb*^*-/-*^ mice compared to WT mice. Total IgM and numbers of ILC2 and B1 cells were comparable in both strains (S6 Fig). Induction of *Il5* mRNA was impaired in LPS_*Ft*_ immunized *Il17rb*^*-/-*^ mice (Fig 6C). Conversely, administration of recombinant IL-5 to *Il17rb*^*-/-*^ mice rescued their IgM_*Ft*_ and IgM_*Ft*_^+^ ASC responses (Fig 6D and 6E). Taken together, these results show that IL-25 released upon LPS_*Ft*_ immunization stimulates ILC2 to secrete IL-5 that supports IgM_*Ft*_ production by B1 cells.

### IL17rb^-/-^ mice are more susceptible to *Ft* infection

**Fig 7:**
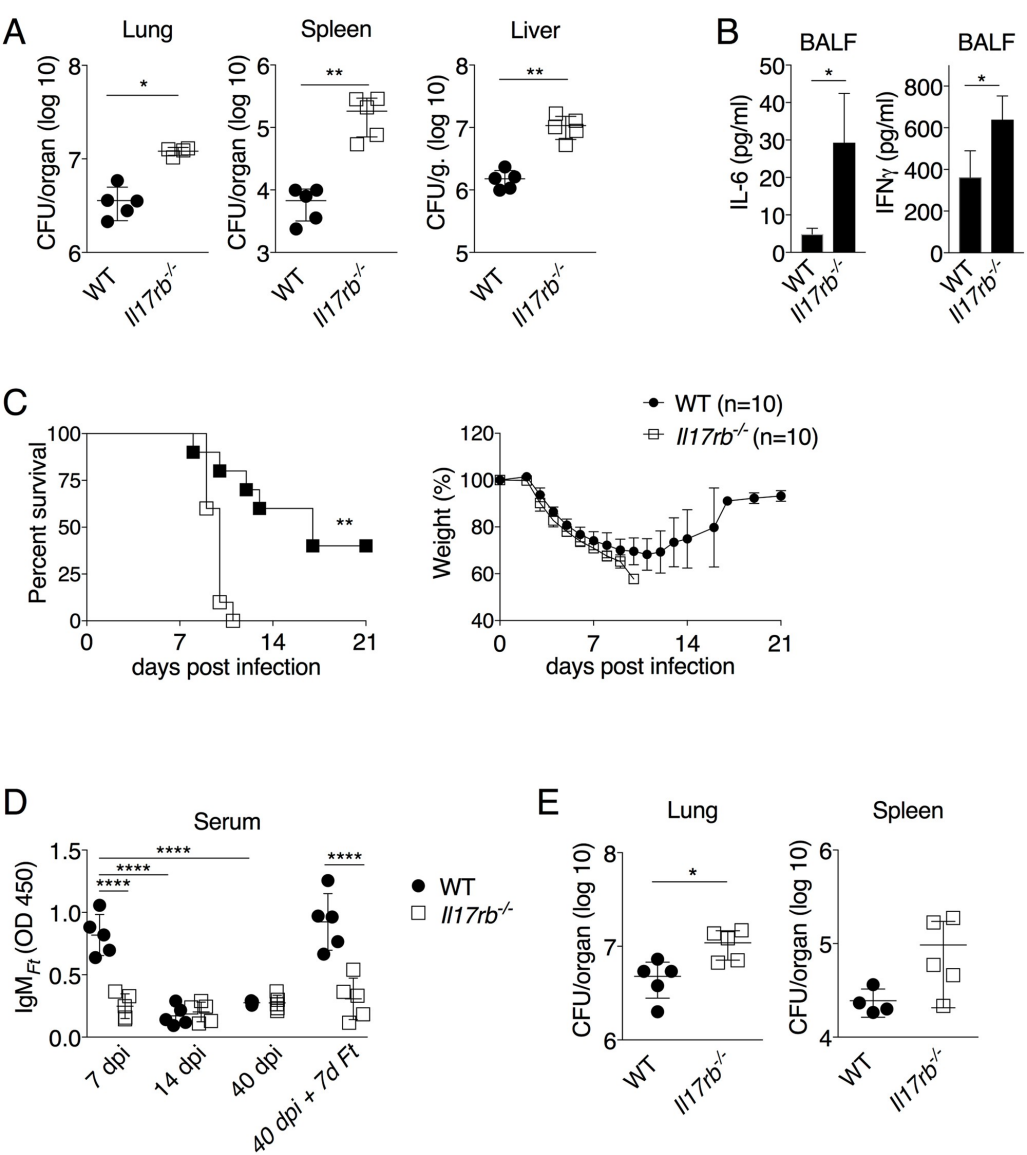
*IL17rb*^*-/-*^ mice are more susceptible to *Ft* infection and fail to develop memory. (A, B) WT B6 or *Il17rb*^*-/-*^ mice were infected with *Ft* (4x10^3^ cfu) and bacteria burden in organs and cytokine levels in BALF were measured 7 days p.i. (C) Mice were infected as in A and their survival and body weight were monitored. (D) WT B6 or *Il17rb*^*-/-*^ mice were immunized i.n. with LPS_*Ft*_ and bled at shown time point. On day 40 p.i. mice were infected with *Ft* (4x10^4^ cfu) and euthanized 7 days later to measure IgM_*Ft*_ and bacteria burden in organs (D). One representative experiment of two. Data are expressed as mean ± SD. A, B, E Mann-Whitney U test; C, Kaplan Meier; D one-way ANOVA Tukey Post-test; E,. *p<0.05, **p<0.01, ****p<0.0001.

We previously showed that passive immunization with IgM_*Ft*_ protects against *Ft* LVS infection [20] suggesting that IgM_*Ft*_ is one of several protective immune mechanisms against tularemia. As shown in Fig 7A, *Il17rb*^*-/-*^ mice i.n. infected with *Ft* had significantly higher bacteria burdens in organs 7 days p.i. compared to WT mice. Reflecting the increased bacteria burden, the level of proinflammatory cytokines IL-6 and IFNγ were significantly higher in *Il17rb*^*-/-*^ mice (Fig 7B). When the survival of intranasally infected mice was monitored, all *Il17rb*^*-/-*^ mice succumbed by day 10 p.i. whereas the mean-time to death for WT mice was significantly longer and 40% of the WT mice survived the infection and gain back weight (Fig 7C). To test whether *Il17rb*^*-/-*^ mice were able to develop IgM memory B1 cells, WT and *Il17rb*^*-/-*^ mice were LPS_*Ft*_-immunized and on day 40 were infected with a higher dose of *Ft* (4x10^5^). As shown in Fig 7D, the level of IgM_*Ft*_ 7 days post-infection (day40+7) rose significantly in WT but not in *Il17rb*^*-/-*^ mice. As a consequence, *Il17rb*^*-/-*^ mice had significantly higher bacteria burden (Fig 7E). We conclude that absence of IL-25 receptor increases susceptibility to *Ft* infection and impairs development of memory B1 cells.

## Discussion

The mechanisms that control generation of antibody against TI antigens by B1 cells are understood to a much lower degree than the responses mediated by B2 cells against proteinaceous T-dependent antigens. Previous works from our and others groups have revealed the critical involvement of B1 cells in the response to infection with *Ft* [14,20]. The results presented here deepen our understanding of this process and shed new light on still unresolved aspects of B1 cell biology. Our data show that B1 cells differentiation into ASC and production of IgM_*Ft*_ can occur in absence of TLR stimulation and depends on IL-5. This response is impaired in absence of ILC2 that are the likely source of IL-5. Importantly, we identify a previously unreported critical role for IL-25 in the production of IgM_*Ft*_ through the stimulation of ILC2. Taken together, these results indicate that the IL-25-ILC2-IL-5 axis plays a crucial role for activation of B1 cells by LPS_*Ft*_.

Although both B1a and B1b cells have been reported to produce IgM in response to infection, it is becoming clear that CD5 expression cannot adequately describe the functional plasticity and heterogeneity of B1 cells [7]. In our experiments, adoptive transfer of purified B1b cells into *Rag1*^*-/-*^ mice was sufficient for production of IgM_*Ft*_ whereas adoptively transferred B1a were found to differentiate into the B1b phenotype that was also associated with IgM_*Ft*_ production. These results are in agreement with a model where, following encounter with the antigen, down-regulation of CD5 expression relieves BCR inhibition and allows B1 cells to become activated, migrate to lymphoid organs, and differentiate into ASC. While this model may apply to B1 cells’ response to several types of infection, it may not explain production of natural IgM. Whether CD5 expression identifies different activation states rather than *bona fide* cell subsets with distinct function remains to be determined.

B1 cells migration to spleen and differentiation into ASC has been shown to be regulated by TLR/MyD88 signaling [40]. In agreement with this notion, we previously showed that production of IgM_*Ft*_ during intranasal *Ft* infection depends on IL-1β and TLR2 signaling [20]. Data presented here also show that migration of B1 cells to spleen is significantly higher in *Ft* infected mice compared to mice immunized with purified LPS_*Ft*_, where TLR stimulation does not occur. However, we found that in the context of immunization with purified LPS_*Ft*,_ production of IgM_*Ft*_ does not require TLR or MyD88-mediated signaling. We believe this apparent discrepancy is due to the fact that during infection the MyD88-dependent inflammation and phagocytes activation are necessary in order to release LPS from the bacterial membrane in amounts sufficient to achieve B1 cells activation. Immunization with purified LPS_*Ft*_ provides enough antigen making the MyD88-dependent step superfluous. Supporting this scenario, immunization with LPS_*Ft*_, either i.n. or i.p., consistently yielded stronger responses than infection. Interestingly, concomitant TLR4 stimulation has been shown to decrease the response to immunization with LPS_*Ft*_ [19], a phenomenon we also observed and that raises important questions regarding the relative role of innate immune signaling versus antigen-specific signals in B1 cells activation. Another important difference between immunization and infection that may be related to stimulation of innate immune pathways was observed during the secondary response. TI antigens were believed for a long time to be unable to generate memory response or long-lived plasma cells. This view is being increasingly challenged by a number of studies [1,3,13]. Our results show that in immunized mice the majority of the IgM_*Ft*_ ASC elicited by the secondary boost immunization was found in the peritoneal cavity rather than the spleen. In mice previously infected, the boost immunization did not increase the number of IgM_*Ft*_ ASC in either peritoneum or spleen. These results suggest that immunization, but not infection, generates a population of LPS_*Ft*_-specific IgM memory B1 cells that resides in the peritoneum and whose reactivation and differentiation into IgM_*Ft*_ ASC no longer depends on spleen-derived cues. It is known that memory B cells differ from naïve B cells in their propensity to migrate and their ability to reside and survive in different anatomical locations [53]. It is presently unclear why infection did not generate the peritoneal IgM memory B1 cells. It is possible that concomitant TLR stimulation, which has been shown to inhibit the response to LPS_*Ft*_ [19], not only impairs the primary B1 response but also the development of B1 memory, as our data suggest. These results challenge the notion that inclusion of adjuvants that stimulate innate immunity invariably results in improved vaccination efficacy. In the case of TI antigens, like LPS_*Ft*_, TLR stimulation may be counter-productive, an idea that has been proposed previously [15] and that will be tested in future studies.

IL-5 is known to regulate B1 cells development and activation [45–48]. Our results are in agreement with this notion and showed that IL-5 was rapidly induced by LPS_*Ft*_ immunization, supported in vitro ASC differentiation, and was required for IgM_*Ft*_ production in vivo. Our results further suggested that ILC2 were the primary source of IL-5 in LPS_*Ft*_ immunized mice. The location of ILC2 at mucosal sites, particularly the airways, and their ability to rapidly produce large amount of type 2 cytokines well position them to be first responders against infection at barrier surface [26]. While most of the studies on ILC2 have been focused on helminths infections and allergy, their role in the response to bacteria infections has been understudied. ILC2 were shown to protect from *Staphylococcus aureus* infection by secreting type 2 cytokines that promoted eosinophilia and reduced neutrophils-mediated damage [54]. The role of ILC2 in regulating the humoral immune response has been investigated only in a few studies. Lung ILC2 were shown to promote proliferation of B1 and B2 cells and production of IgM and IgA against T-independent antigens and to support B1 cells self-renewal [55,56]. These responses were dependent on IL-5, similarly to what we observed. Our results indicate that ILC2 are essential for the production of IgM_*Ft*_ and are therefore critically involved in the generation of a protective humoral response against *Ft*.

At least two subsets of ILC2 have been identified: natural ILC2 (nILC2), that are strongly responsive to IL-33 stimulation, and inflammatory ILC2 (iILC2), that do not express the IL-33 receptor ST2 but respond strongly to IL-25 and express the activation marker KLRG1. The fact that IgM_*Ft*_ production was dependent on IL-25 but not Myd88, on which IL-33 signaling depends, suggests that iILC2 are the subset involved in this response. This conclusion is further supported by the observation that immunization increases expression of the IL-5-reporter gene of Red5 mice in the KLRG1^+^ ILC2 population. Redundancy of MyD88 also suggests that IL-18, another cytokine that strongly activates ILC2, is not involved in IgM_*Ft*_ generation. Interestingly, activation of B1 cells during filarial nematode pleural infection was shown to depend on a IL-33-ILC2-IL-5 axis [24] that appears analogous to the one we describe here for the first time but with some important differences: the subtype of ILC2 activated (IL-33-responsive nILC2 rather than IL-25-responsive iILC2) and the fact that B1 cells and IgM production remained localized to the respiratory lymphoid structures, as observed during influenza infection, in contrast to systemic IgM and B1 cells migration to spleen observed in our model.

IL-25 (IL-17E), a member of the IL-17 cytokine family, is an alarmin released in response to stress and tissue damage by a number of cell types [27] including epithelial cells, mast cells, basophils, eosinophils, and, most prominently, Tufts cells, a chemosensory cell type found in the lung and intestinal epithelium [57]. IL-25 is a potent activator of iILC2 and T_h_2 cells and numerous studies have shown that it is critical for responses against helminths and during allergic reactions by inducing secretion of type 2 cytokines IL-5 and IL-13. However, IL-25’s role during bacterial infection has remained, until now, unexplored. Our results are the first to indicate that it plays a critical role in the B1 cells response to LPS_*Ft*_. Mice deficient in IL-17RB, the IL-25 receptor subunit expressed at high level by iILC2, had impaired response to primary or secondary LPS_*Ft*_ immunization. This phenotype was not due to insufficient number of ILC2 but rather to impaired IL-5 production. Accordingly, administration of IL-5 rescued IgM_*Ft*_ production in *Il17rb*^*-/-*^ mice. Finally, our results show that *Il-17rb*^*-/-*^ mice were more susceptible to *Ft* infection, the first evidence that IL-25 protects against a respiratory bacterial infection.

T-independent antigens are classically divided into TI-1, which can activate TLR, and TI-2 whose repetitive structure is sufficient to trigger BCR clustering and achieve B cells activation [58]. While it is likely that the ability of LPS_*Ft*_ to stimulate B1 cells is related to its repetitive structure, it is unclear how this bacterial product can trigger IL-25 release. Whether and how LPS_*Ft*_ stimulates Tuft cells or other cell types to release IL-25 remains to be determined.

The involvement of the IL-25-ILC2-IL-5 axis in the response to other TI antigens is an important issue that should be addressed in future studies. NP-Ficoll, a model TI-2 antigen, stimulates B1b cells and elicits TI memory responses [59]. These features, and its inability to stimulate TLR, are shared by LPS_*Ft*_ suggesting that the IL-25-ILC2-IL-5 axis may also contribute to the humoral response against NP-Ficoll. The role of this axis in the activation of B1 cells by TI antigens of other microbes may be more complex to determine. Several studies have examined B1 cell activation in the context of infection but not immunization with purified microbial Ag. Not surprisingly, the response to infection was often found to depend on TLR signaling, like in the case of *B*. *hermsii*, *Salmonella*, and influenza virus [60–62]. It is conceivable that, in absence of TLR stimulation, activation of B1 cells comes to rely more heavily on the IL-25-ILC2-IL-5 axis, as in the case of LPS_*Ft*_. Pneumococcal polysaccharides are recognized by B1b cells [16] and are the main component of vaccines against *S*. *pneumoniae*. Interestingly, it has been shown that the humoral immune response elicited by pneumococcal polysaccharides-based vaccines, including the commercial Pneumovax 23 and Prevnar vaccines, is dependent on the presence of associated TLR ligands [63] underscoring how much we still need to learn about the mechanism of their immunogenicity.

In summary, our result indicate that immunization with purified LPS_*Ft*_ triggers an IL-25-ILC2-IL-5 axis that controls production of IgM_*Ft*_ by B1 cells and provide long-term protection from infection with *Ft*. Vaccination strategies that target this pathway may improve the effectiveness of immunization against TI antigens.

## Materials and methods

### Ethics statement

All the animal experiments described in the present study were conducted in strict accordance with the recommendations in the *Guide for the Care and Use of Laboratory Animals* of the National Institutes of Health. All animal studies were conducted under protocols approved by the Rosalind Franklin University of Medicine and Science Institutional Animal Care and Use Committee (IACUC) (protocol # B12-07). All efforts were made to minimize suffering and ensure the highest ethical and humane standards.

### Mice

C57BL/6J, *Tlr2*^*-/-*^, *Myd88*^*-/-*^, *Rag1*^*-/-*^, *Red5(Il5*^*-/-*^*)*, *and Kit*^*W-sh*^ mice were purchased from Jackson lab. *Il17rb*^*-/-*^ mice were obtained from Hiroshi Watarai (Kanazawa University). All mouse strains were on C57BL/6 genetic background and were bred under specific pathogen-free conditions in our facility. Age-(8–10 weeks old) and sex-matched animals were used in all experiments. Experimental groups were composed of 3–5 mice. All the animal experiments described in the present study were conducted in strict accordance with the recommendations in the *Guide for the Care and Use of Laboratory Animals* of the National Institutes of Health. All animal studies were conducted under protocols approved by Institutional Animal Care and Use Committees of the Rosalind Franklin University of Medicine and Science (protocol # B20-10, B18-11). All efforts were made to minimize suffering and ensure the highest ethical and humane standards.

### LPS_*Ft*_ purification

LPS was purified from mid logarithmic phase liquid culture of *Francisella tularensis* LVS using the phenol-based LPS Extraction Kit (iNtRON Biotechnology). The LPS preparation was then treated with RNase A, DNase I, and Protienase K, re-extracted, precipitated, and resuspended in Tris NaCl at a concentration of 100 μg/ml. Absence of TLR-agonist contaminants was confirmed by stimulating bone marrow derived macrophages and TLR2, TLR4/MD2-reporter HEK293 cell lines.

### Bacteria culture, mice infection, and measurement of bacteria burden in organs

For all experiments the *Francisella tularensis* LVS was used. Bacteria were grown in MH broth (Muller Hinton supplemented with 0.1% glucose, 0.1% cysteine, 0.25% ferric pyrophosphate, and 2.5% calf serum) to mid-logarithmic phase, their titer was determined by plating serial dilutions on complete MH agar, and stocks were maintained frozen at -80°C. No loss in viability was observed over prolonged storage. For infections, frozen stocks were diluted in sterile PBS to the desired titer. Aliquots were plated on complete MH agar to confirm actual CFU. Mice were anesthetized with isoflurane using a Surgivet apparatus and 50 μl of bacteria suspension were applied to the nare. Organs aseptically collected were weighted and homogenized in 1 ml PBS containing 0.5% saponin and 3% BSA. Serial dilutions were plated on complete MH agar plates using the Eddy Jet Spiral Plater (Neutec). Bacterial colonies were counted 48 hours later using the Flash & Grow Automated Bacterial Colony Counter (Neutec).

### Mice immunization and treatments

To immunize mice, 100 ng of LPS_*Ft*_ were diluted in sterile PBS and administered intranasaly (50 μl) or intraperitoneally (200 μl). Although both routes elicited specific B1 cell responses, i.p. immunization consistently yielded more robust serum antibody titers and number of spleen ASC. In contrast, i.n. immunization was preferable for measurement of responses localized to the lung like those concerning ILC2 and IL-5 and IL-25. In some experiments, mice received the following treatments: recombinant mouse IL-5, or IL-25 (2.5 μg, i.n. on the day of experiment, i.p. on the following three days); anti-CD90.2 or isotype-matched antibody (BioXCell, 200 μg, i.p. 24 hours before start of experiment, on then on day 0, 1, 3, 5); FTY720 (100 μg, i.p., on the day of experiment and then on day 1, 3, 6).

### Determination of antibody titers and cytokine levels by ELISA

Blood was collected aseptically from the submandibular vein. BALF were collected from euthanized mice by intratracheal injection and aspiration of 1 ml PBS. The peritoneal and thoracic cavities were washed twice with 2 ml PBS/Pen/Strep. *Ft* LPS-specific immunoglobulin levels in serum or lavages were measured by ELISA. Serial dilutions were plated in 96 wells plates coated with purified LPS_*Ft*_ (100 ng/ml). HRP-conjugated goat anti-mouse IgM or IgG_3_ (Southern Biotech Associates, Birmingham, AL) was added followed by TMB substrate and measurement of absorbance at 450 nm. Cytokine levels were measured by ELISA using the following paired antibodies kits: mIL-5, mIL-6, mIL-25, mIFNγ, (Invitrogen). For IL-5 and IL-25 detection, BALF and thoracic lavage were concentrated 10X using Millipore spin filtration devices 3 KDa cutoff.

### ELISPOT

Multiscreen 96 well Filter plates (Millipore) were coated overnight with LPS_*Ft*_ (50 ng/ml) and blocked in 1% BSA for two hours. Single cell suspensions from spleen, peritoneum, or thoracic cavity were plated (5x10^5^ or 5x10^4^ cells/well) in RPMI1640/10% FCS, Pen/Strep/Amphothericin. Two days later, plates were washed and LPS_*Ft*_-specific spots revealed with HRP-conjugated rat anti-mouse IgM and TMB substrate. Each well was photographed and spots counted. For in vitro experiments, peritoneal and thoracic cells were enriched by negative selection for B cells using the Pan B cell isolation kit (StemCell) and were cultured for three days +/- IL-5 (50 ng/ml) and then used to seed ELISPOT.

### Flow cytometry

Lung was minced and digested with collagenase IV and DNAse I for 1 hours in RPMI Pen/Strep. Single cells were filtered with 70 μm mesh and red blood cells lysed. Cells obtained from peritoneum, thoracic lavage, or spleen, were resuspended in FACS buffer (1% BSA, 0.05% NaN3 in PBS, Fc block CD16/32) and stained with the following antibodies: FITC-conjugated Ter119, CD3ε, CD4, CD11c, TCRβ, TCRγδ, CD5; APC-Cy7-conjugated CD11b, CD19, Ly6G, NK1.1; CD45-PerCP-Cy5.5, CD278-APC, KLRG1-BV421, Thy1.2-Biot, CD5-PE, IgM-PE-Cy7, IgD-APC, CD23-BV421, CD43-PerCP-Cy5.5. B1 cells are defined as CD19^+^, IgM^high^, IgD^-/low^ CD43^+^, CD23^-^, CD5^+/-^. ILC2 are defined as lineages negative (Ter119, CD3ε, CD4, CD11c, TCRβ, TCRγδ, CD5CD11b, CD19, Ly6G, NK1.1) and positive for CD45, CD278, KLRG1, Thy1.2. Isotype controls were used to set the gates. Data were acquired with a BD LSR II flow-cytometer (BD biosciences) and analyzed with FlowJo 10.4 software (Treestar Inc).

### B1 B cells isolation, cell sorting purification, and adoptive transfer

The peritoneal cavity was washed twice with 2 ml PBS/Pen/Strep. B cells were enriched using the Pan B cells kit (StemCells Technologies). Cells were stained with CD19, IgM, IgD, CD43, CD23, and CD5 to label B1a and B1b cells. Both populations were purified using a BD FACS Aria II U cell sorter. The purity of B1 subsets was routinely higher than 96%. B1 cells (5x10^5^) were adoptively transferred into *Rag1*^*-/-*^ mice by i.p. injection. Four weeks later mice were used for experiments.

### RT PCR

Total RNA was isolated from lung using Trizol. One μg of total RNA was treated with DNase I and cDNA was generated using random hexamers and SuperScript III First-Strand Synthesis System (Invitrogen). Quantitative PCR was performed using PowerUp SYBR Green Master Mix (Applied Biosystems,) using 1 μl of cDNA template per reaction. The following primers were used: mIL-5 F TCA GGG GCT AGA CAT ACT GAA G; R CCA AGG AAC TCT TGC AGG TAA T; b-Actin F GGC TGT ATT CCC CTC CAT CG; R CCA GTT GGT AAC AAT GCC ATG T. Values were calculated via the 2^-ddCt^ method for relative fold change in gene expression where ddCt is calculated by subtracting the dCt (gene of interest Ct–bActin Ct) of each experimental sample from the averaged dCt of the calibrator samples (naive mice).

### Statistical analysis

All data were expressed as mean ± SD. Statistical analysis was performed with GraphPad Prism 7 using Mann-Whitney U test, unpaired student *t*- test, one-way ANOVA Tukey Post-test, or Kaplan-Meier as specified in figure legends. Significance was set at *p*≤0.05.

## Supporting information

S1 Fig Related to Fig 1. (PDF)

S2 Fig Related to Fig 2. (PDF)

S3 Fig Related to Fig 3. (PDF)

S4 Fig Related to Fig 4. (PDF)

S5 Fig Related to Fig 5. (PDF)

S6 Fig Related to Fig 6. (PDF)

S1 Data Related to Fig 1. (PZFX)

S2 Data Related to Fig 2. (PZFX)

S3 Data Related to Fig 3. (PZFX)

S4 Data Related to Fig 4. (PZFX)

S5 Data Related to Fig 5. (PZFX)

S6 Data Related to Fig 6. (PZFX)

S7 Data Related to Fig 7. (PZFX)
