# Supplementary material for: Critical role of IL-25-ILC2-IL-5 axis in the production of anti-Francisella LPS IgM by B1 B cells
Source: PLoS Pathog. 2021 Aug 27;17(8):e1009905. doi: 10.1371/journal.ppat.1009905 (PMC8428711; doi:10.1371/journal.ppat.1009905)

S1 Fig, Related to Fig 1. (A) Total IgM for figure 1A. (B) WT B6 mice were immunized with shown amount of LPS_{Ft}. Seven days post-immunization IgM_{Ft} was measured in serum. (C, D) Total number of B1 cells and representative flow plots and gating strategy used to identify B1a and B1b cells for figure 1A. (E) Representative ELISPOT images for figure 1B.

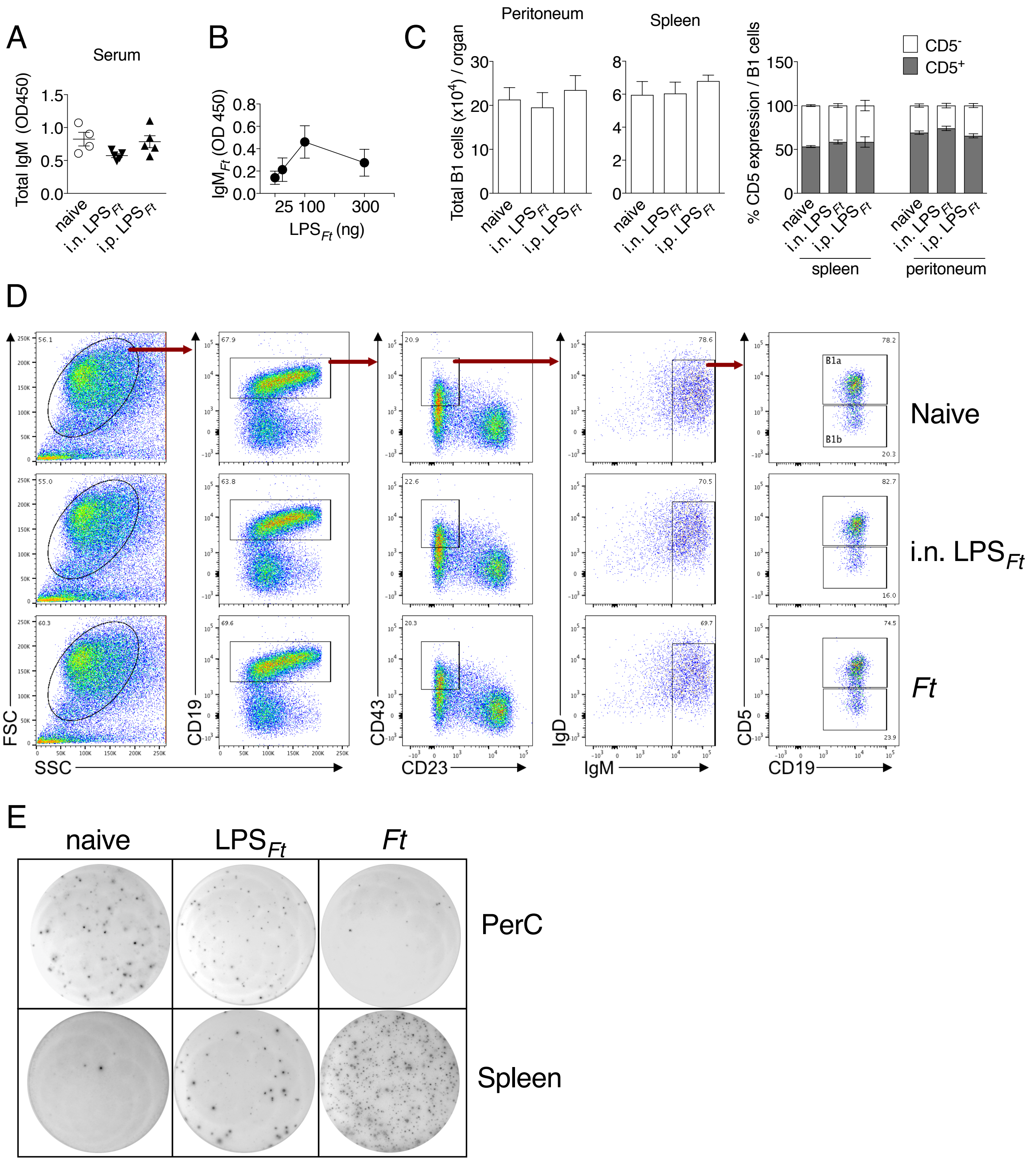

Supplement: S1 Fig — (PDF) [file ppat.1009905.s001.pdf]
