# Supplementary material for: Critical role of IL-25-ILC2-IL-5 axis in the production of anti-Francisella LPS IgM by B1 B cells
Source: PLoS Pathog. 2021 Aug 27;17(8):e1009905. doi: 10.1371/journal.ppat.1009905 (PMC8428711; doi:10.1371/journal.ppat.1009905)

S2 Fig, Related to Fig 2. Gating strategy used to sort purify B1a and B1b transferred into *Rag1*$^{-/-}$ mice in figure 2. Top row, Pan B enriched; middle row purified B1a; bottom row, purified B1b.

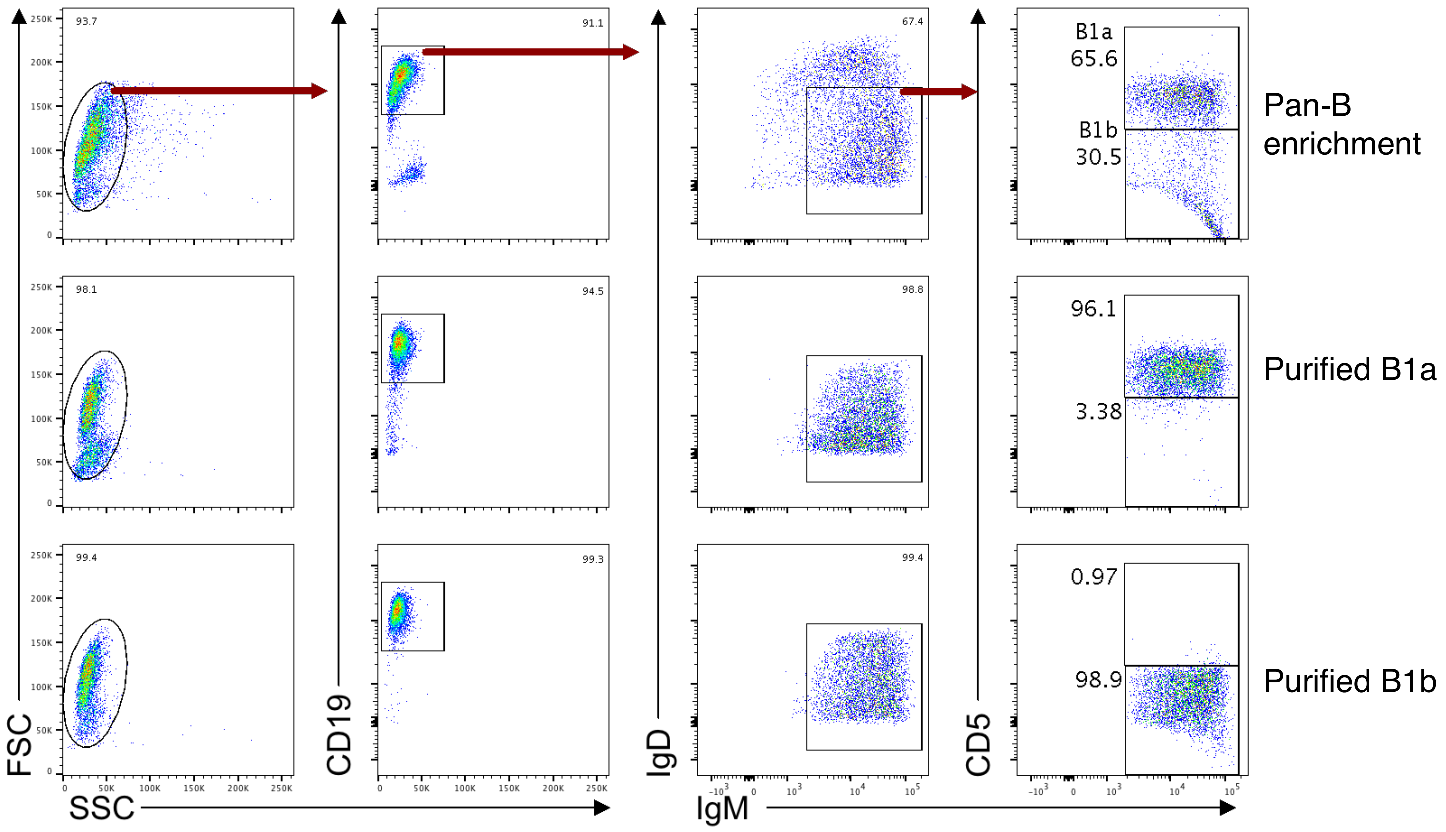

Supplement: S2 Fig — (PDF) [file ppat.1009905.s002.pdf]
