# Supplementary material for: Critical role of IL-25-ILC2-IL-5 axis in the production of anti-Francisella LPS IgM by B1 B cells
Source: PLoS Pathog. 2021 Aug 27;17(8):e1009905. doi: 10.1371/journal.ppat.1009905 (PMC8428711; doi:10.1371/journal.ppat.1009905)

S3 Fig, Related to Fig 3. (A) Total IgM for figure 3B. (B) Representative flow plots used to identify B1 cells in WT and *Il5*$^{-/-}$ mice of figure 3C. (C) Representative flow plots and gating strategy used to identify KLRG1^{+} ILC2 in WT and *Il5*$^{-/-}$ mice of figure 3C.

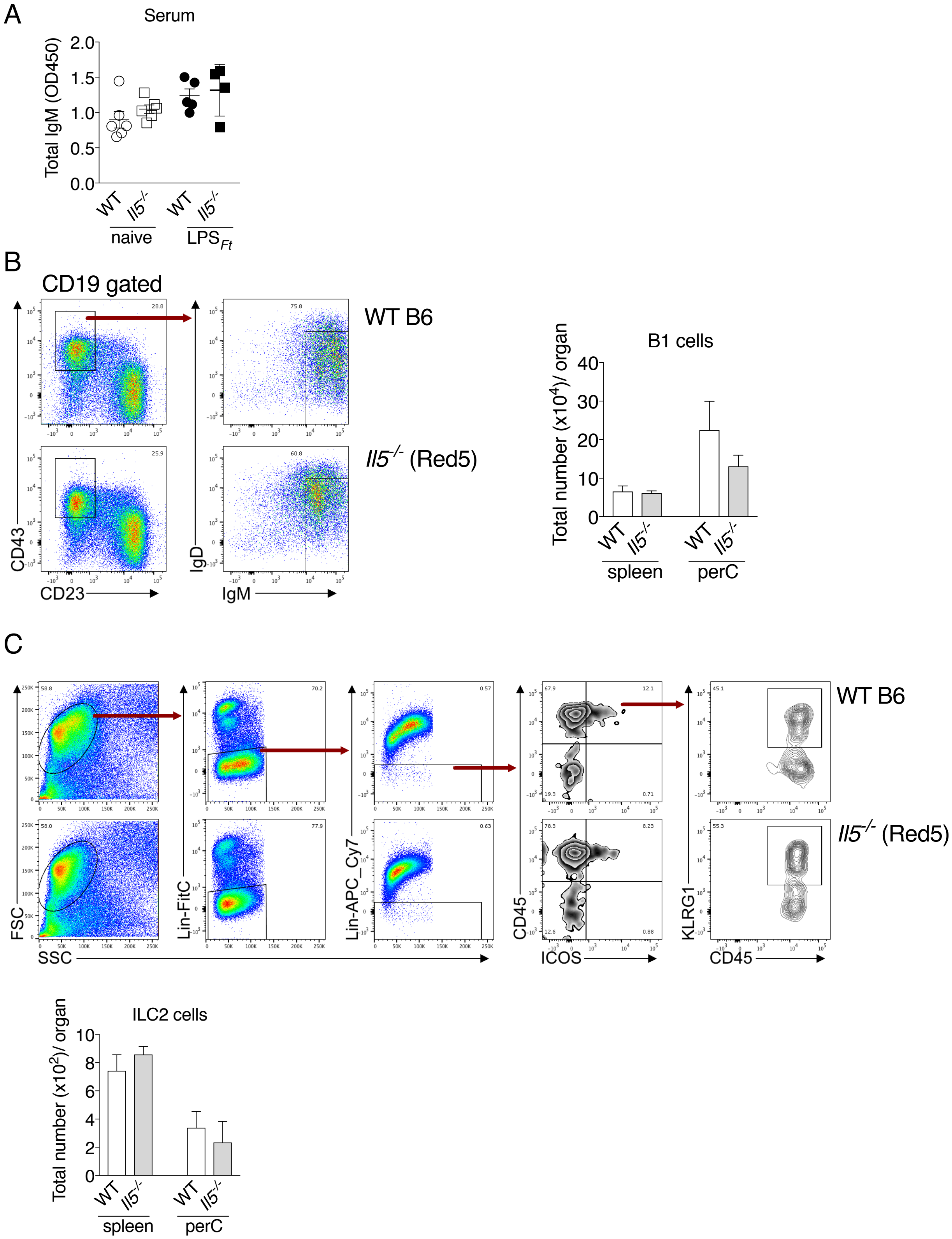

Supplement: S3 Fig — (PDF) [file ppat.1009905.s003.pdf]
