# Supplementary material for: Critical role of IL-25-ILC2-IL-5 axis in the production of anti-Francisella LPS IgM by B1 B cells
Source: PLoS Pathog. 2021 Aug 27;17(8):e1009905. doi: 10.1371/journal.ppat.1009905 (PMC8428711; doi:10.1371/journal.ppat.1009905)

S4 Fig, Related to Fig 4. (A) Frequency of ILC2 in depleted WT B6 mice of figure 4C and representative flow plots. (B) Total IgM and numbers of B1 cells and numbers of IgM$_{Ft}$ ASC for figures 4B, C. (C) Representative flow plots and gating strategy used to identify Red5$^+$ KLRG1$^+$ ILC2 of figure 4E.

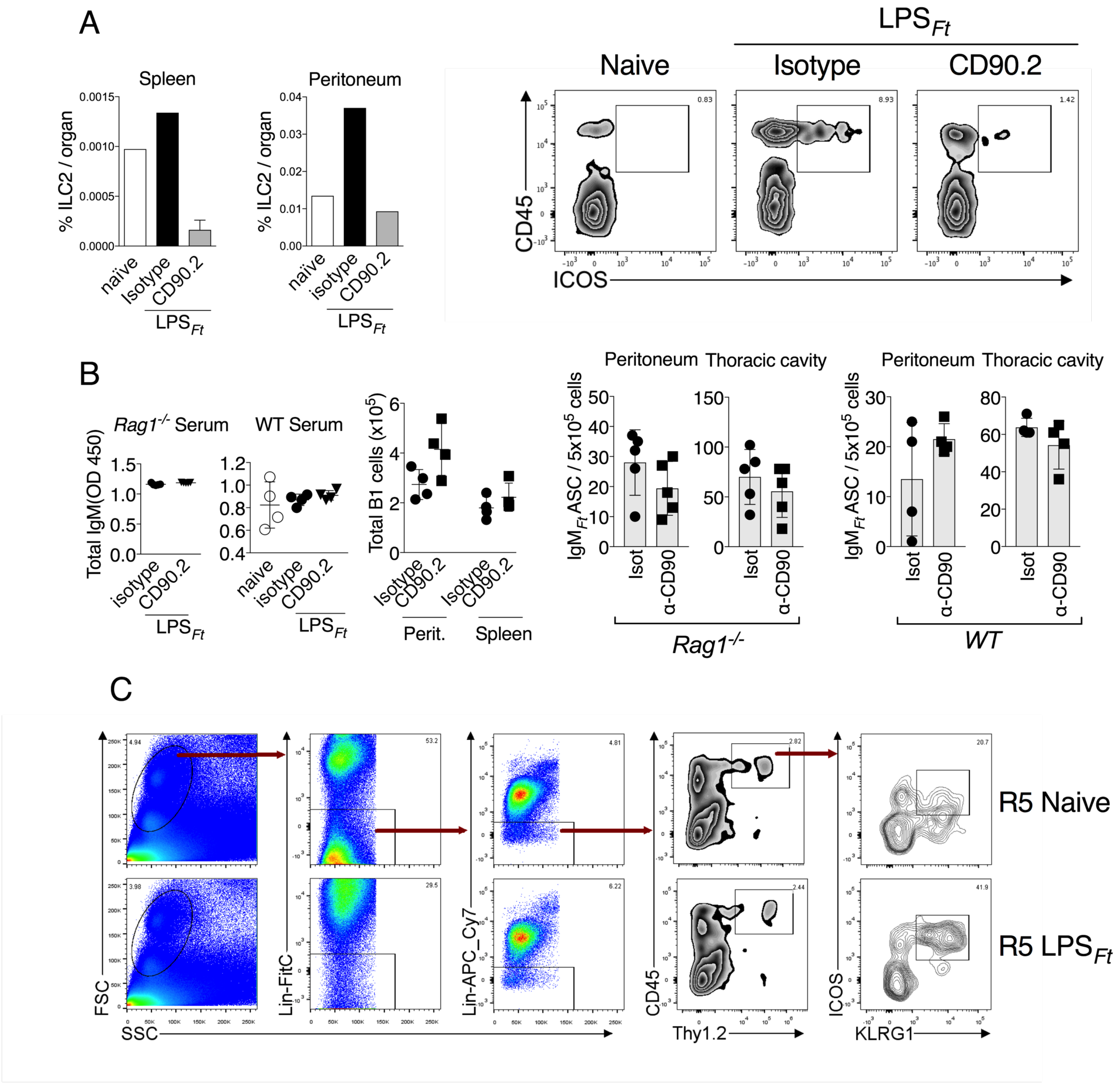

Supplement: S4 Fig — (PDF) [file ppat.1009905.s004.pdf]
