# Supplementary material for: Critical role of IL-25-ILC2-IL-5 axis in the production of anti-Francisella LPS IgM by B1 B cells
Source: PLoS Pathog. 2021 Aug 27;17(8):e1009905. doi: 10.1371/journal.ppat.1009905 (PMC8428711; doi:10.1371/journal.ppat.1009905)

S6 Fig, Related to Fig 6. (A) Total IgM for figure 6A. (B, C) Total numbers and representative flow plots used to identify of B1 cells and KLRG1^{+} ILC2 in WT B6 and *Il17rb*$^{-/-}$ mice of figure 6B.

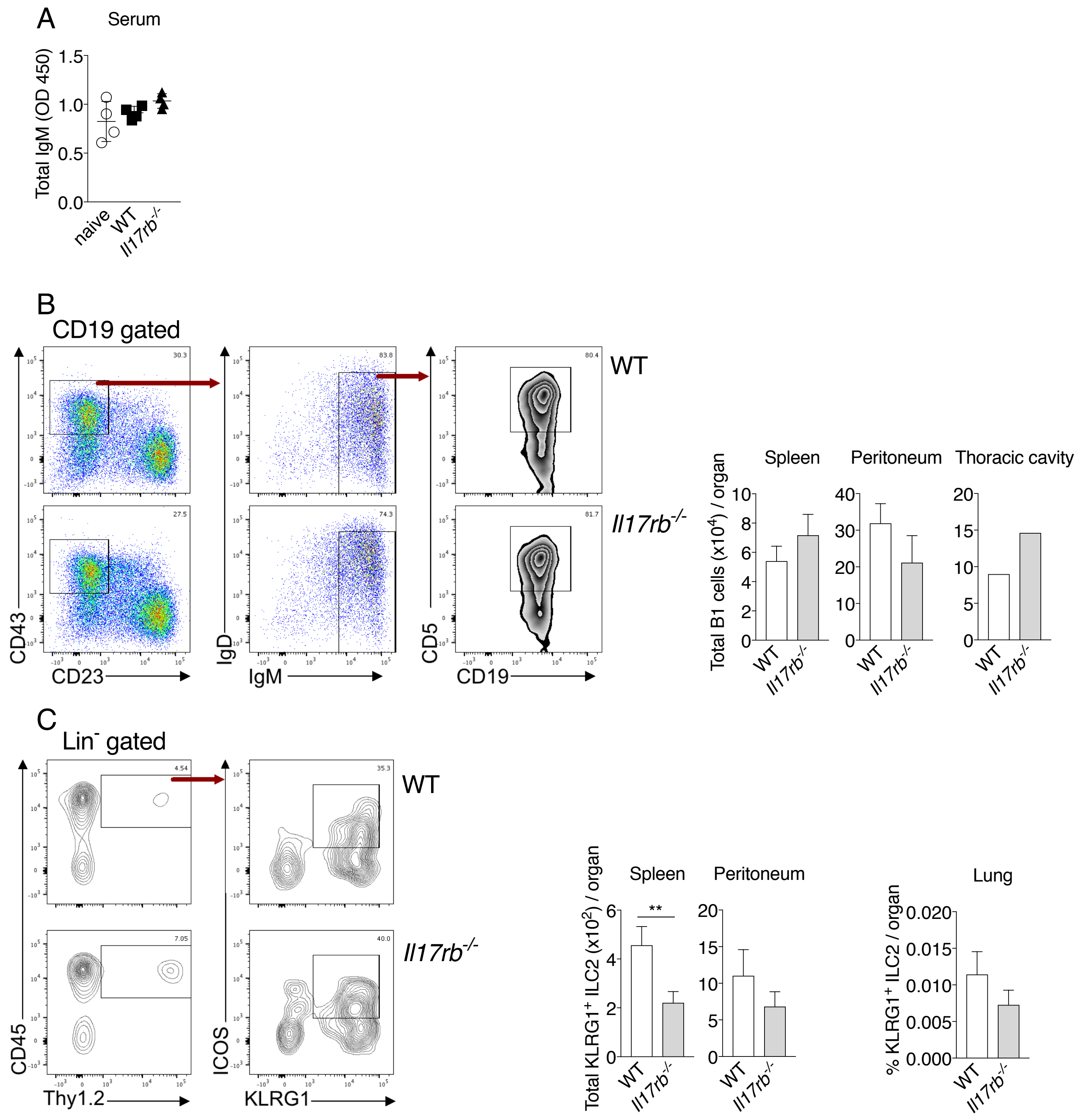

Supplement: S6 Fig — (PDF) [file ppat.1009905.s006.pdf]
